# In vitro and in vivo evaluation of the radiosensitizing effect of a selective FGFR inhibitor (JNJ-42756493) for rectal cancer

**DOI:** 10.1186/s12885-015-2000-8

**Published:** 2015-12-16

**Authors:** Maud Verstraete, Annelies Debucquoy, Annelies Gonnissen, Ruveyda Dok, Sofie Isebaert, Ellen Devos, William McBride, Karin Haustermans

**Affiliations:** 1 Department of Oncology, Laboratory of Experimental Radiotherapy, KU Leuven, Herestraat 49, 3000 Leuven, Belgium; 2 Radiation Oncology, University Hospitals Leuven, Herestraat 49, 3000 Leuven, Belgium; 3 Department of Radiation Oncology, David Geffen School of Medicine, UCLA, 200 UCLA Medical Plaza, Suite B265, Los Angeles, CA 90095-6951 USA

**Keywords:** Colorectal cancer, Cancer therapy, FGFR, In vitro, In vivo, Radiotherapy

## Abstract

**Background:**

We examined the anti-tumor effect and radiosensitizing potential of a small molecule inhibitor of fibroblast growth factor receptor (FGFR) in colorectal cancer (CRC) in vitro and in vivo.

**Methods:**

Effects of in vitro drug treatment on cell survival, proliferation, FGFR signaling, cell cycle distribution, apoptosis and radiosensitivity were assessed using various CRC cell lines with FGFR wild type (Caco2 and HCA7) and FGFR2 amplification (HCT116, NCI-H716). In vivo tumor responses to FGFR inhibition with and without radiation therapy were evaluated by growth delay assays in two colorectal xenograft mouse models (NMRI nu/nu mice injected with NCI-H716 or CaCo2 cells). Mechanistic studies were conducted using Western blot analysis, immunohistochemistry and qPCR.

**Results:**

In the tested cell lines, the FGFR inhibitor (JNJ-42756493) was effective in vitro and in vivo in CRC tumors with highest expression of FGFR2 (NCI-H716). In vitro, cell proliferation in this line was decreased, associated with increased apoptotic death and decreased cell survival. In vivo, growth of NCI-H716 tumors was delayed by 5 days by drug treatment alone, although when drug delivery was stopped the relative tumor volume increased compared to control. The FGFR inhibitor did not radiosensitize NCI-H716 tumors either in vitro or in vivo.

**Conclusions:**

Among tested CRC cell lines, the growth inhibitory activity of this FGFR inhibitor was evident in cell lines with high constitutive FGFR2 expression, suggesting that FGFR addiction may provide a window for therapeutic intervention, though caution is advised. Preclinical study with NCI-H716 and Caco2 tumor demonstrated that continued presence of drug could be essential for tumor growth control, especially in cells with aberrant FGFR expression. In the tested set-up, the inhibitor showed no radiosensitizing effect.

**Electronic supplementary material:**

The online version of this article (doi:10.1186/s12885-015-2000-8) contains supplementary material, which is available to authorized users.

## Background

The standard treatment for patients with rectal cancer is chemoradiotherapy followed by surgery, but 30 % of these patients develop local and distant recurrences [1]. Therefore, an intensification of the preoperative treatment, particularly through the use of molecular targeted agents, could be beneficial. Fibroblast growth factors (FGFs) and their receptors are recognized oncogenes associated with a variety of cancers, including colorectal cancer (CRC), and are therefore attractive therapeutic targets.

The mammalian FGF family comprises 18 ligands, which act through 4 FGFRs (FGFR1, FGFR2, FGFR3 and FGFR4) [2, 3]. Binding to the receptors causes activation of two key downstream pathways: the mitogen-activated protein kinase-extracellular signal-regulated kinase (MAPK-ERK) and phosphoinositide3-kinase (PI3K)-AKT pathway [3], which mediate several physiological responses during embryonic development and in the adult organism, including angiogenesis, tissue repair and hematopoiesis [4].

Dysregulated expression of many FGFs and all four FGFRs has been reported in CRC, especially for FGFR2 [5–11]. The effectiveness of FGFR2-targeting therapy for CRC has been demonstrated in vitro and in vivo illustrating the potential of FGFR2 as novel molecular target for CRC [7].

The effects of FGFR pathway inhibition in combination with radiotherapy have not been investigated extensively but inhibition of the cell cycle and angiogenesis could augment the tumor response [12, 13], as could drug-induced impairment of DNA repair [14].

The main objective of our study was to mechanistically evaluate the effects of a pan-FGFR tyrosine kinase inhibitor (JNJ-42756493) with and without radiotherapy. Our hypothesis was first evaluated in vitro in several established human colorectal cell lines. Since the in vitro setup does not allow us taking into account the influence of the tumor-micro environment, the most promising human colorectal cell lines were in a second step injected in nude mice (NMRI nu/nu) allowing us to evaluate the in vivo efficacy of this treatment scheme.

## Methods

### Cells and cell culture

Several human colorectal cell lines were used: HCT116, HCA7 (European Collection of Cell Culture, Salisbury, UK), Caco2 and NCI-H716 (American Type Culture Collection, Manassas, VA, USA). HCT116 cells were maintained at 37 °C in a humidified incubator with 5 % carbon dioxide/95 % air atmosphere in McCoys5A + GlutaMAX (l-alanyl-l-glutamine), HCA7 in Dulbecco’s Modified Eagle’s Medium and Caco2 and NCI-H716 cells in RPMI 1640 + GlutaMAX-I medium, all supplemented with 10 % Fetal Bovine Serum (Invitrogen, Carlsbad, California, USA). For HCA7 and Caco2 cells 1 % sodiumpyruvate (Invitrogen) was added. Results on HCT116 and HCA7 cells are in Additional file 1: Figure e1 and Additional file 2: Figure e2.

### FGFR inhibitor

An ATP-competitive small molecule tyrosine kinase inhibitor against FGFR1-4 (JNJ-42756493) was provided by Janssen Pharmaceutica. JNJ-42756493 is a potent, oral pan-FGFR tyrosine kinase inhibitor with half-maximal inhibitory concentration values in the low nanomolar range for all members of the FGFR family (*FGFR1* to *FGFR4*), with minimal activity on vascular endothelial growth factor receptor (VEGFR) kinases compared with FGFR kinases (approximately 20-fold potency difference) [15]. The drug was dissolved in dimethylsulfoxide (Sigma, St. Louis, MO, USA) prior to dilution for in vitro use and in cylodextrin as vehicle for in vivo experiments. Vehicle controls were used where appropriate.

### Cell viability

The effect of varying drug concentrations on cell growth and survival was evaluated at 72 h using sulforhodamine B (SRB) assay for the adherent cells (HCT116, HCA7, Caco2) [16] and trypan blue dye exclusion for the suspension cells, NCI-H716.

### Flow cytometry

Flow cytometry experiments were performed using BD FACS Canto (Becton Dickinson, Franklin Lakes, NJ, USA) and analyzed using BD FACS Diva Software.

#### Cell cycle

After treatment with varying drug doses, for 24 or 72 h, cells were fixed with 70 % ethanol and stained with 10 μg/ml propidium iodide (Sigma)/100 μg/ml RNaseA (Invitrogen) solution.

#### Cell proliferation

Cells were labeled with 75 μM bromodeoxyuridine (BrdU) (Sigma) and counterstained for DNA with propidium iodide after 24 h drug treatment. Anti-Brdu-FITC antibody was added (Becton Dickinson, San Jose, CA, USA) after DNA denaturation with 2 N HCl/Triton x-100 and neutralization with sodium borate.

#### Apoptosis

Apoptosis was determined using the Annexin-V-FLUOS detection kit (Roche, Hague-Road, IN, USA) 72 h after drug treatment.

### Clonogenic assays

Cells were incubated in the presence of drug at various concentrations. After incubation, cells were irradiated in suspension with 2, 4 or 6 Gy on a clinical linear accelerator (Varian, Palo Alto, CA, USA) using 6 Mega Volt photons and seeded in triplicate into 10-cm culture dishes (Caco2) or diluted into 0.3 % soft agar onto 0.5 % solidified agar in 10-cm culture dishes (NCI-H716). After 12 or 21 days incubation for Caco2 and NCI-H716 cells respectively, colonies were fixed and stained with crystal violet. Colonies (≥50 cells) were counted with ColCount™ colony counter (Oxford Optronic, Oxford, UK) and survival fractions were calculated.

### Mice

Animal experiments were approved by the animal ethics committee of the Catholic University Leuven and performed in a licensed A1 laboratory by staff with the required FELASA certificates taking into account the 3R’s of the use of animals in research. Since we need immune deficient mice to develop our xenograft models with human cell lines, NMRI nu/nu female mice (Janvier, Saint-Berthevin, France) were used. At the start of the experiment, the mice were on average 7 weeks (6–8 weeks) and had an average weight of 29.5 g (22–33). The mice were housed in individually ventilated cages with a maximum of 4 mice per cage in a room with a controlled light/day cycle and controlled temperature.

#### Sample size calculation

The drug tested in the current proposal will be considered effective if it can prolong the growth delay of the tumors with a minimum of 5 days. Taking into account the effect of 5 days, a standard deviation of 2 days, an α value of 0.05 and a power of 0.80, the minimal sample size is three. We divided each treatment group into 2 subgroups; one group to assess the immediate molecular effects (TP1) and one group for the tumor growth delay assay (TP2). Therefore, a minimum of 6 mice per group was included in the experiment. All in vivo experiments were repeated twice. The data shown in the manuscript are representative of one of two independent experiments.

#### Xenograft models

The mice were inoculated in both flanks with 3*10^6^ of Caco2 or 5*10^6^ of NCI-H716 cells in Matrigel (BD Biosciences, Bedford, MA, USA) subcutaneously in both flanks. When tumors were on average 100 mm^3^, the mice were randomized in 4 treatment groups (control, radiotherapy, FGFR inhibitor, radiotherapy + FGFR inhibitor). Each mice had two tumours (one in each flank) and the tumours were used as unit for the statistical analyses. The mice in the different groups were matched for tumour size to allow a good comparison of the treatment effect in all groups. The characteristics of the mice can be found in Tables 1 and 2 for the NCI-H716 and CaCo2 experiment respectively.

**Table 1:** Mice characteristics at start of the treatment experiment (NCI-H716 xenograft model)

		Group 1	Group 2	Group 3	Group 4	TOTAL
Number of animals		6	7	7	7	27
Number of tumours		12	14	14	14	54
Weight (g)	Mean	30	29	29	29	29
	Range	28–33	27–32	28–31	28–31	27–33
Tumour size (mm^3^)	Mean	100	95	97	96	97
	Range	26–181	25–165	40–271	39–182	25–271
Treatment given		vehicle	5Gy + vehicle	FGFR inhibitor	FGFR inhibitor + 5 Gy

**Table 2:** Mice characteristics at start of the treatment experiment (CaCo2 xenograft model)

		Group 1	Group 2	Group 3	Group 4	TOTAL
Number of animals		6	6	7	6	25
Number of tumours		12	12	14	12	50
Weight (g)	Mean	29	31	29	31	30
	Range	22–32	28–33	24–31	29–32	22–33
Tumour size (mm^3^)	Mean	166	168	167	169	168
	Range	88–255	79–199	52–259	112–218	52–259
Treatment given		vehicle	5Gy + vehicle	FGFR inhibitor	5 Gy + FGFR inhibitor	

#### Treatment

The FGFR inhibitor was dissolved in cyclodextrin and administered at a dose of 40 mg/kg as suggested by Janssen Pharmaceutica. The vehicle used was also cyclodextrin. Both the vehicle and the drugs were administered in the morning three times a week for three weeks by gavage. At the end of the second week (day 12) of drug treatment, the tumors were irradiated with 5 Gy (NCI-H716) or 10 Gy (CaCo2) with a Clinical Linear Accelerator (Varian, Palo Alto, CA, USA) using 16 Mega electron volt (MeV). During the irradiation, the mice were anesthetized with Nembutal (CEVA, Brussels, Belgium).

#### Experimental outcomes

The experimental outcomes included a growth delay assay to assess the growth inhibiting effect as well as the radiosensitizing effect of the drug and the assessment of molecular changes in the tumours after treatment. Treatment response was evaluated by tumor growth delay using thrice-weekly caliper measurements. Mice were sacrificed by cervical dislocation either at the end of the anti-FGFR treatment (time-point 1 (TP1)) (5 mice/group) or when tumors were >2000 mm^3^ (time-point 2 (TP2)) (5–7 mice/group). Thirty minutes before sacrifice, mice were injected with 60 mg/kg pimonidazole (Hypoxyprobe, Burlington, MA, USA). Part of each tumor was fixed in formalin and embedded in paraffin for immunohistochemistry and part was snap frozen for protein and mRNA analyses.

### Irradiation

Irradiation was delivered to the cells or tumor site with a clinical linear accelerator. In vitro, the cells were irradiated with 6 MeV photons (dose rate of 2.4Gy/min) for irradiation of cells in suspension (2, 4, 6Gy). In vivo, the tumors were irradiated with 16 MeV electrons with a dose rate of 3Gy/min. For the NCI-H716 xenograft model, a single dose of 5 Gy was used. Tumors of the CaCo2 xenograft model were irradiated with a single dose of 10Gy. The in vitro and in vivo radiation setups were calculated by the department of Radiation Oncology and recalculated on a regular basis.

### Gene expression

Quantitative PCR (qPCR) was used to measure copy numbers for all four FGFRs in cell lines and FGFR2, FGF1, FGF2, VEGF-A, PlGF, VEGFR1 and VEGFR2 of mouse and human origin in xenografted tumors. RNA was isolated by the Qiagen RNeasy Mini Kit (Qiagen, Hilden, Germany). RNA was reverse transcribed using the SuperScript VILO cDNA synthesis Kit (Invitrogen) followed by qPCR reactions with the Lightcycler 480 (Roche, Mannheim, Germany). Reactions were carried out on cDNA from cultured cells with Lightcycler 480 Sybr Green I master (Roche) and self-designed primers (IDT, Coralville, IA, USA). Reactions on cDNA from tumors were performed with Taqman Fast Universal PCR Master Mix (Applied Biosystems, Foster city, CA, USA) using premade probes (Applied Biosystems/IDT) (Additional file 3: Table e1).

### Western blotting

Cell and tumor lysates were prepared in lysis buffer as described before [17]. Total protein amount was measured using the Bradford method (Bio-Rad, Hercules, CA, USA). 10–30 μg of proteins was subjected to electrophoresis on NuPage gels (Invitrogen). Immunoblotting was performed with antibodies recognizing phospho-FGFR (1:1000), AKT (1:1000), phospho-AKT (1:500), phospho-ERK (1:500), PARP (1:1000), cleaved PARP (1:1000), or β-actin (1:1000) from Cell Signaling Technology (Beverly, MA) and FGFR2 (1:400) (R&D, Minneapolis, MN, USA) or ERK2 (1:1000) (Santa Cruz Biotechnology, Dallas, TX, USA), followed by incubation with the appropriate horseradish peroxidase-conjugated secondary antibodies (1:3000) (Cell Signaling; GE Healthcare, Little Chalfont, UK).

### Immunohistochemical staining and analysis

After antigen retrieval and blocking, tumor sections were incubated overnight at 4 °C with anti-pimonidazole (1/400, Hypoxyprobe), anti-caspase-3 (ready to use, Biocare Medical) or anti-CD31 (1/200, BC Biosciences); or for 30 min at room temperature with anti-Ki67 (ready to use, Thermo Scientific). Appropriate secondary antibodies followed by 3.3’-diaminobenzidine (DAB) substrate (DAKO, Glostrup, Denmark) were used to visualize antigen presence. Protocol details are in Additional file 4: Table e2.

Tumor hypoxic and apoptotic fractions were determined by the percentage of pimonidazole- and caspase-3 positive cells respectively, the latter using the method of Going [18]. Ki67 positive nuclei were counted as an index of proliferation and CD31 used to determine the number of blood vessels per field for 20 fields per tissue specimen as a measure of micro vessel density (MVD).

### Statistical analysis

Statistical analyses used a one-way analysis of variance with Tukey’s multiple comparison tests for in vitro comparisons and a Mann–Whitney *U* test for in vivo tumor growth delay.

For the in vivo experiments, the single tumours were used as unit of analysis. Immunohistochemical and qPCR data from in vivo studies were analyzed using a two-tailed student’s *t*-test when the data complied with the conditions of normality and equal variance. Under other conditions, comparisons were carried out by nonparametric analysis using the Mann–Whitney rank-sum test. The Kolmogorov-Smirnov method was used to test for normality. A significance level of *p* = 0.05 was used in all cases. Statistics were calculated using Statistica software 12 (StatSoft Inc, Tulsa, OK).

## Results

### Anti-tumor activity in vitro

**Fig. 1:**
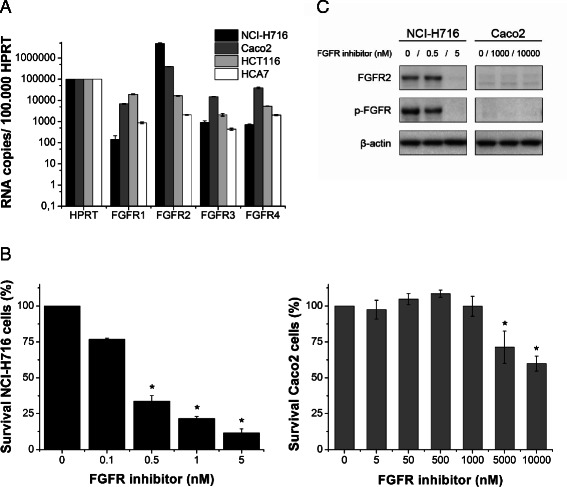
Anti-tumor activity in vitro. **a** Quantification of FGFR mRNA expression by qPCR. HPRT copy number was used to normalize the data. Data = means ± SEM of two independent experiments performed in duplicate. **b** Effect of different concentrations FGFR inhibitor for 72 h incubation on cell survival. Data = means ± SEM from three independent experiments performed in triplicate. ∗Significantly different from control conditions at the appropriate drug concentrations (*p* < 0.05; Tukey). **c** Immunoblot analysis of FGFR2 after 72 h treatment. β-actin was used as loading control. Blots shown are representative for one of two independent experiments

All four FGFRs were detected by qPCR in all CRC cell lines in vitro (Fig. 1a). Highest expression, as compared to the household gene HPRT, was for the FGFR2 gene in NCI-H716 (48 fold) and Caco2 cells (4 fold) (*p* < 0.05) (Fig. 1a). In agreement with these findings, FGFR inhibition significantly decreased cell growth and survival of NCI-H716 cells at concentrations of ≧ 0.5nM (*p* < 0.05), whereas ≧ 5000nM was required for all other cell lines, including Caco2 (Fig. 1b, Additional file 1: Figure e1A). In NCI-H716 cells FGFR2 mRNA expression increased in a dose-dependent manner after drug treatment (Additional file 2: Figure e2A) while protein expression, which was detectable only in this cell line, decreased (Fig. 1c). Also p-FGFR protein levels decreased upon treatment, confirming FGFR inhibition. No other changes in mRNA or protein expression were noted in any of the cell lines for any of the FGFRs (Additional file 2: Figure e2A, Additional file 1: Figure e1B and Fig. 1c). Based on these data, NCI-H716 and Caco2 cell lines were chosen for further in vitro and in vivo experimentation.

### In vitro effects on cell proliferation, apoptosis and radiosensitivity

**Fig. 2:**
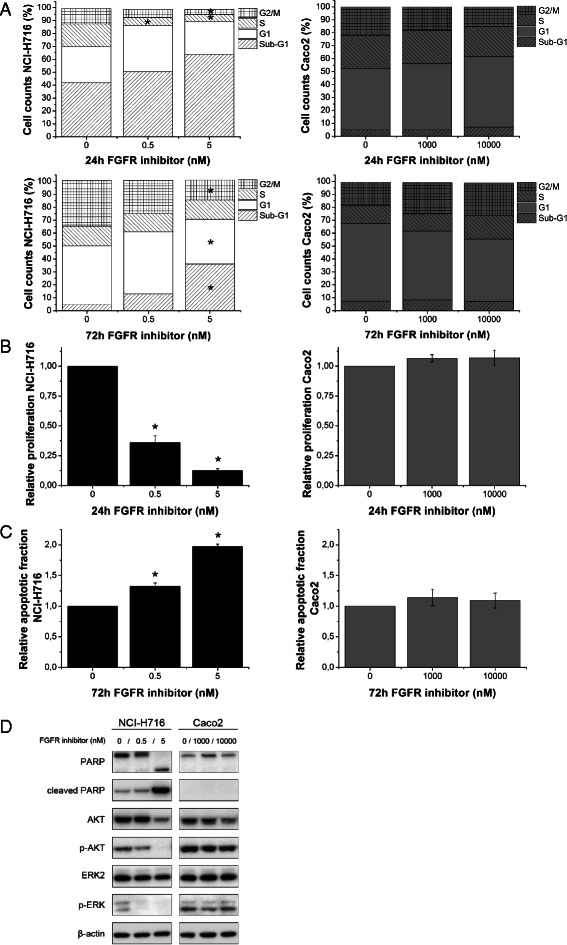
In vitro effect on cell cycle distribution, proliferation and apoptosis. **a** Cell-cycle distribution of propidium stained cells after 24 (up) and 72 h (below) drug incubation. **b** BrdU incorporation after 24 h drug incubation. **c** Annexin-V detection after 72 h drug incubation. Data = means ± SEM from three independent experiments performed in triplicate. ∗Significantly different from control conditions (*p* < 0.05; Tukey). **d** Immunoblotting for (cleaved)PARP and downstream signaling molecules after 72 h drug incubation. β-actin served as loading control. Blots shown are representative for two independent experiments

**Fig. 3:**
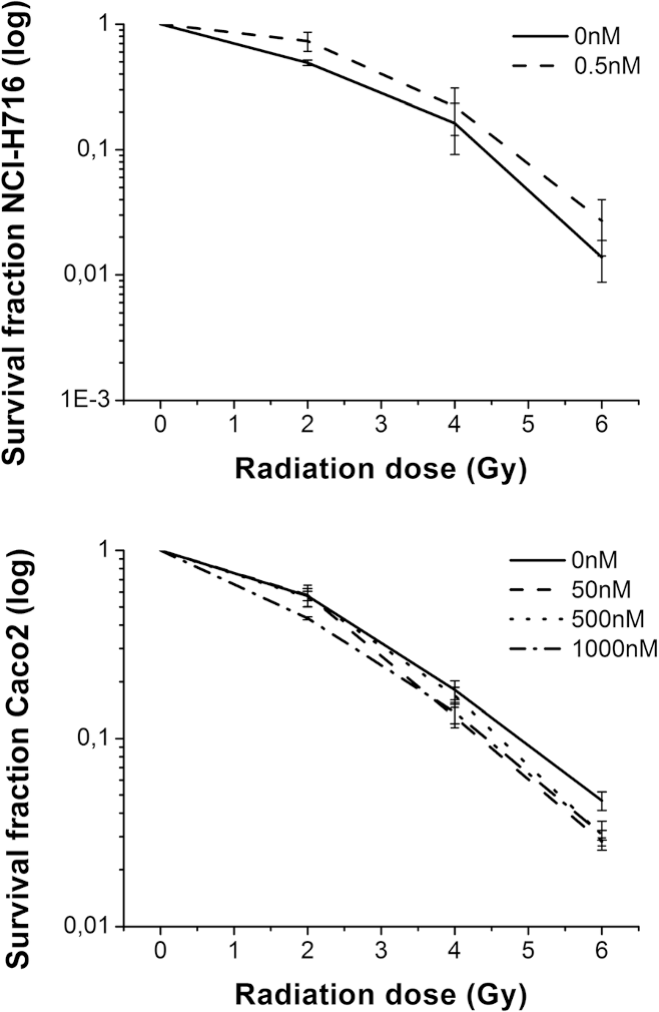
Radiosensitizing effect in vitro. Radiosensitizing effect of the indicated concentrations FGFR inhibitor on NCI-H716 (24 h) and Caco2 (72 h) cells tested by soft agar and colony formation assay, respectively. Data = means ± SEM of at least three independent experiments performed in triplicate

The FGFR inhibitor induced significant changes in cell cycle distribution, proliferation and apoptosis in the NCI-H716, but not the Caco2, cell line (Fig. 2). The G2/M and S subpopulations were significantly decreased after 24 h incubation with 5nM drug (*p* < 0.05; Fig. 2a) and this was confirmed by decreased S-phase BrdU labeling (*p* < 0.05) (Fig. 2b). The increase in apoptosis suggested by the changes in the sub-G1 population (Fig. 2a) was confirmed by Annexin-V labeling at 0.5nM (*p* < 0.05) (Fig. 2c). Molecular analyses by western blotting were consistent with these findings, with drug-induced PARP cleavage, and inhibition of p-AKT and p-ERK in NCI-H716 but not Caco2 cells (Fig. 2d). FGFR inhibitor concentrations that affected FGFR2 receptor expression and cell growth (Fig. 1) did not significantly affect clonogenic survival following irradiation of NCI-H716 or Caco2 cells with 2, 4 or 6 Gy (Fig. 3).

### Xenograft growth delay

**Fig. 4:**
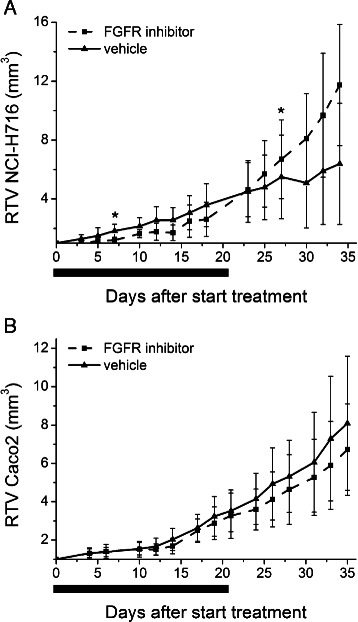
Anti-tumor activity in vivo. Growth curves of mice bearing NCI-H716 (**a**) and Caco2 (**b**) xenograft tumors (▪). Control tumor-bearing animals received vehicle (▴). Relative tumor volumes (RTV) are shown. The line under the graph represents the period of treatment. All data points are mean ± STDEV of at least 10 tumors per treatment group. NCI-H716 data are representative of one of two independent experiments. ∗Significantly different from each other (*p* < 0.05; Mann–Whitney *U* test)

To assess the growth inhibiting effect of the drug, we compared the relative tumour volumes of xenografted mice in the control group (group 1) and the group receiving only the FGFR inhibitor (group 3) (Tables 1 and 2). Relative tumor volume of NCI-H716 tumors was delayed by drug treatment by 5 days (*p* < 0.05), with an increase in volume of on average 24.1 % of pretreatment values at 1 week (−15.9–92.73 %) while the control group increased by 88.2 % (−30.3–257.2 %) (*p* < 0.05) (Fig. 4). However when drug treatment was stopped, the relative tumor volume significantly increased between day 25 and 30 compared to vehicle treatment. The average increase in tumor volume one week after the end of drug treatment was 129.4 % (63.9–208.4 %) for the experimental group and 31.1 % (−25.2–89.1 %) for the control group (*p* < 0.05). The data of the CaCo2 experiment are not shown since no significant growth delay was observed in Caco2 treated tumors. No adverse events were observed in any of the experimental groups.

### Mechanism of action of FGFR inhibition in vivo

**Fig. 5:**
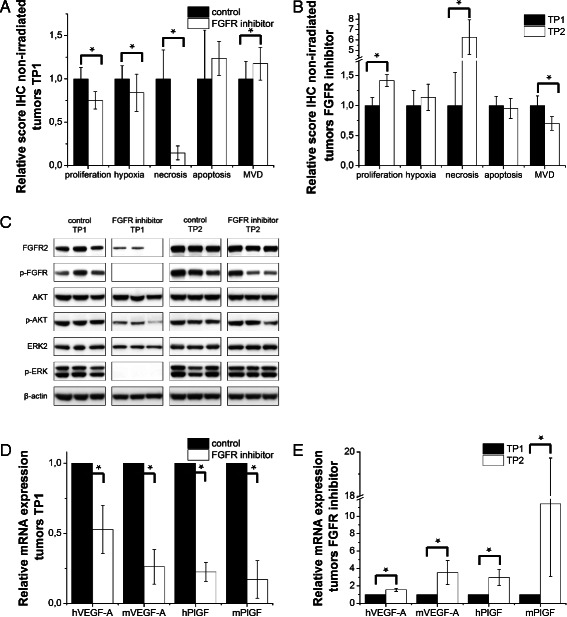
Reversible in vivo action. NCI-H716 tumors were isolated after drug treatment (TP1) and at the end of the experiment (TP2). **a** Effect of the FGFR inhibitor on proliferation, hypoxia, necrosis, apoptosis and micro vessel density (MVD). **b** Comparison between TP1 and TP2 (= effect drug cessation). Columns indicate mean ± STDEV of at least 20 tumor sections per treatment group. ∗Significantly different from one another (*p* < 0.05; two-tailed student’s *t*-test). **c** Western blot for indicated proteins. β-actin served as loading control. Shown blots are from three tumors from different mice per group. **d** mRNA expression in isolated tumors at TP1. **e** Comparison of mRNA expression levels between TP1 and TP2. Data = means ± SEM of three independent experiments. ∗Significantly different from each other (*p* < 0.05; two-tailed student’s *t*-test)

Tumors harvested immediately after the end of FGFR inhibitor treatment (TP1) showed a significant reduction in proliferation, hypoxia and necrosis as compared to control tumors (*p* < 0.05) while apoptosis tended to be increased, as did MVD (*p* < 0.05) (Fig. 5a). At later time points (TP2), these effects disappeared as illustrated by an increase in proliferation (*p* < 0.05), hypoxia (N.S.) and necrosis (*p* < 0.05) and decrease in MVD (*p* < 0.05) while the apoptotic index was unaltered (Fig. 5b).

Western blotting of tumor extracts at TP1 showed a marked decrease in p-FGFR, p-ERK, and p-AKT in tumors treated with inhibitor (Fig. 5c). Human and murine VEGF-A and PlGF mRNA expression was also decreased (*p* < 0.05) (Fig. 5d). These effects were lost at TP2 (Fig. 5c), when in fact VEGF-A and PlGF mRNA expression was markedly increased in the drug-treated group (*p* < 0.05) (Fig. 5e).

In contrast with the in vitro mRNA expression data, tumors harvested at TP1 showed reduced human and murine FGFR2 expression levels as compared to control tumors (N.S.) (Additional file 2: Figure e2B).

### Combination treatment in vivo

**Fig. 6:**
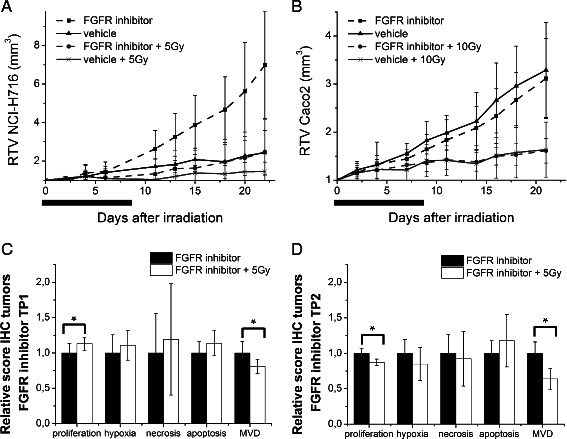
Radiosensitizing effect in vivo. Mice bearing NCI-H716 (**a**) and Caco2 (**b**) xenograft tumors were treated with FGFR inhibitor with or without a single dose of radiotherapy at day 12 of the anti-FGFR treatment (▪ ●). Control tumor-bearing animals received vehicle (▴ x). Relative tumor volumes (RTV) are shown. The line under the graph represents the period of drug treatment. All data points are mean ± STDEV of at least 10 tumors per treatment group. NCI-H716 data are representative for one of two independent experiments. **c**, **d** Effect of irradiation in treated tumors after drug treatment (TP1) and at the end of the experiment (TP2). Columns indicate mean ± STDEV of at least 20 tumor sections per treatment group. ∗Significantly different from one another (*p* < 0.05; two-tailed student’s *t*-test)

To assess the radiosensitizing effect of the drug, the tumor growth of mice in the treatment group with only irradiation (group2) were compared with the group receiving both radiation therapy and the FGFR inhibitor (group 4) (Tables 1 and 2). While irradiation with 5 Gy (NCI-H716) or 10 Gy (CaCo2) slowed tumor growth in both models, the addition of FGFR inhibitor did not radiosensitize either (Fig. 6a, b). On the other hand irradiation with 5 Gy prevented the relative accelerated growth of NCI-H716 tumors following drug withdrawal. No adverse events were observed in any of the experimental groups.

Immunohistochemistry confirmed the absence of any drug-radiation interaction. Hypoxia, necrosis and apoptosis were the same in the two cohorts. Proliferation at TP1 showed a small but significant increase in the irradiated group (*p* < 0.05), while MVD was decreased (*p* < 0.05) (Fig. 6c). The effects of irradiation in slowing the regrowth of drug treated tumors (Fig. 6a), were reflected in a decreased proliferation index at TP2 (*p* < 0.05) (Fig. 6d).

## Discussion

Targeting pathways that are dysregulated in cancer promise to improve tumor control and increase patient survival, but it is an approach that may generally have to be combined with conventional cytotoxic therapies like radiotherapy. Different inhibitors against the FGFR pathway have been tested as monotherapy or in combination with other targeted drugs for different cancer types with promising preclinical and clinical results [19–23]. However, the radiosensitizing effect of FGFR inhibition has not been extensively investigated.

In the tested cell lines, this FGFR inhibitor showed potent and selective anti-tumor activity against the cell line with known FGFR2 amplification (NCI-H716), compared with other cell lines with low/not detected protein expression of FGFR2 (Caco2, HCT116 and HCA7). The NCI-H716 cell line displayed high constitutive p-FGFR expression both in vitro and in vivo that was strongly inhibited by the drug. This is in agreement with other reports [21, 24–30] and with the first clinical data obtained with this inhibitor [15]. Proliferation of NCI-H716 cells was abolished, consistent with blockage of the ERK pathway, followed by dramatic increase in apoptosis and significant decrease in cell survival, in agreement with recent published findings where FGFR inhibition indeed exerted pro-apoptotic effects in vitro [29, 30]. These events were reflected at the molecular level by an increase in the apoptotic marker cleaved PARP and inhibition of the pro-survival AKT pathway. In agreement with earlier data with other FGFR specific inhibitors, FGFR inhibition in the NCI-H716 xenograft model resulted initially in tumor growth delay with decreased tumor cell proliferation and inhibition of ERK activation, which is known to be a major downstream target of activated FGFRs [20, 30, 31].

However, when the drug was withdrawn, the ratio of NCI-H716 tumor volume in vivo accelerated dramatically. This was reflected in an increase in relative proliferation compared to controls, the magnitude of which have outstripped angiogenesis as seen in an increase in the ratio of expression of angiogenic factors, decreased MVD and increased necrosis. Based on the in vitro expression data, this relatively accelerated proliferation may have been due to the fact that drug treatment increased FGFR2 mRNA expression while decreasing FGFR2 signaling. The pathway may therefore have been primed cells for accelerated recovery when the drug was withdrawn. In vivo qPCR data is inconsistent with this reasoning with a trend showing a decreased FGFR2 mRNA expression in treated tumors. However, tumoral human FGFR2 expression was much lower than levels detected in the cell line, which might indicate that stromal cells and necrotic tissue in tumors might dilute the signals, possibly explaining this contradiction. Very detailed kinetic analyses may be needed to fully evaluate this hypothesis in absence of any obvious gross effects on the molecular pathways in this study.

In spite of the major effects of the drug on tumor cell proliferation and survival, it did not radiosensitize tumors in vitro or in vivo. Several mutually agonistic and antagonistic factors may be operating in these complex systems. Cell cycle analysis showed that FGFR inhibition leads to a decrease of cells in the G2/M phase, being the most radiosensitive phase of the cell cycle [32], but also a decrease in radioresistant S phase cells. The decrease in tumor proliferation upon FGFR inhibition could hamper the efficacy of the radiation. Similar data have been described by our group with the EGFR inhibitor cetuximab [33]. It should be noted that even these low doses of radiation that were used effectively abolished the relatively accelerated proliferation that followed drug withdrawal. This was associated with effects on tumor cell proliferation and a decrease in MVD, which may have been due to the well-known effect of irradiation on angiogenesis [34]. However it is clear from our in vivo experiment that in the current set-up the FGFR inhibitor does not has a radiosensitizing effect in the CRC cell lines tested. It would be interesting to determine whether another treatment scheme where FGFR inhibition is started after, and not before, irradiation would be a more effective therapy by inhibiting angiogenesis and tumor cell repopulation.

We also have to be aware of some limitations of the study. In our study we focused only on CRC cell lines and not on other tumor types, such as endometrial, gastric and breast cancer, were deregulation of the FGFR pathway has been shown to be implicated in cancer [3]. Also within our CRC cell lines tested, only one of them showed aberrant FGFR expression. Consequently we do not know the effect of the drug on CRC harboring other FGFR deregulations apart from FGFR2 overexpression. Furthermore, our in vivo experiments were performed in mice with a deficient immune system. Therefore the effect that the immune system could have in the response to this combined treatment was not taken into account.

Further research on a larger set of cancer cell lines in needed. Also combining the drug with other targeted agents and chemotherapeutics could be interesting.

## Conclusions

In summary, the FGFR inhibitor used in this study mediated effective cytotoxicity both in vitro and in vivo, but only in cells with aberrant FGFR2 expression [35]. These results underline the dependency of cancer cells upon oncogenic FGFRs which provides a therapeutic opportunity for selective intervention by FGFR inhibitors. Proper patient selection based on FGFR2 status of the tumor will be critical when testing the inhibitor in future clinical trials. However, at least for this agent, the continued presence of drug seems essential and its absence may cause accelerated tumor regrowth. Based on our data, this inhibitor does not augment the cytotoxicity of radiotherapy, but radiotherapy may prevent accelerated tumor regrowth in CRC cell line. Further investigations into how best to optimize their delivery with conventional therapies are needed.

## Additional files

Additional file 1: Figure e1. Effect on cell survival and protein levels in HCA7 and HCT116 cells. (A) Effect of different concentrations FGFR inhibitor for 72 h incubation on cell survival determined by sulforhodamine B assay. Data = means ± SEM from three independent experiments performed in triplicate. *Significantly different from control conditions at the appropriate drug concentrations (*p* < 0.05; Tukey). (B) Immunoblot analysis of FGFR2 and downstream signaling molecules after 72 h treatment. β-actin was used as a loading control. Blots shown are representative for two independent experiments. (PDF 1053 kb)
Additional file 2: Figure e2. Effect on FGFR mRNA levels. (A) Quantification of FGFR mRNA expression in NCI-H716, Caco2, HCT116 and HCA7 cells after 72 h drug incubation. Data = means ± SEM of two independent experiments performed in duplicate. (B) Quantification of FGFR2 mRNA expression in NCI-H716 tumors isolated after drug treatment (TP1). Data = means ± SEM of three independent experiments. HPRT copy number was used to normalize the data. *Significantly different from control conditions at the appropriate drug concentrations (*p* < 0.05; Tukey). (PDF 4011 kb)
Additional file 3: Table e1. Primer sequences, primer probes and cycling conditions used for qPCR of different genes. (DOCX 15 kb)
Additional file 4: Table e2. Specifications antigen retrieval, blocking step and antibodies used for immunohistochemical staining. (DOCX 15 kb)

## Abbreviations

BrdU: Bromodeoxyuridine
CRC: Colorectal cancer
ERK: Extracellular signal-regulated kinase
FGF: Fibroblast growth factor
FGFR: Fibroblast growth factor receptor
Gy: Gray
IHC: Immunohistochemistry
MVD: Micro vessel density
SRB: Sulforhodamine B
TP: Time-point
