# Supplementary material for: In vitro and in vivo evaluation of the radiosensitizing effect of a selective FGFR inhibitor (JNJ-42756493) for rectal cancer
Source: BMC Cancer. 2015 Dec 16;15:946. doi: 10.1186/s12885-015-2000-8 (PMC4682227; doi:10.1186/s12885-015-2000-8)
Supplement: Additional file 1: Figure e1. — Effect on cell survival and protein levels in HCA7 and HCT116 cells. (A) Effect of different concentrations FGFR inhibitor for 72 h incubation on cell survival determined by sulforhodamine B assay. Data = means ± SEM from three independent experiments performed in triplicate. *Significantly different from control conditions at the appropriate drug concentrations (p < 0.05; Tukey). (B) Immunoblot analysis of FGFR2 and downstream signaling molecules after 72 h treatment. β-actin was used as a loading control. Blots shown are representative for two independent experiments. (PDF 1053 kb) [file 12885_2015_2000_MOESM1_ESM.pdf]

Figure e1

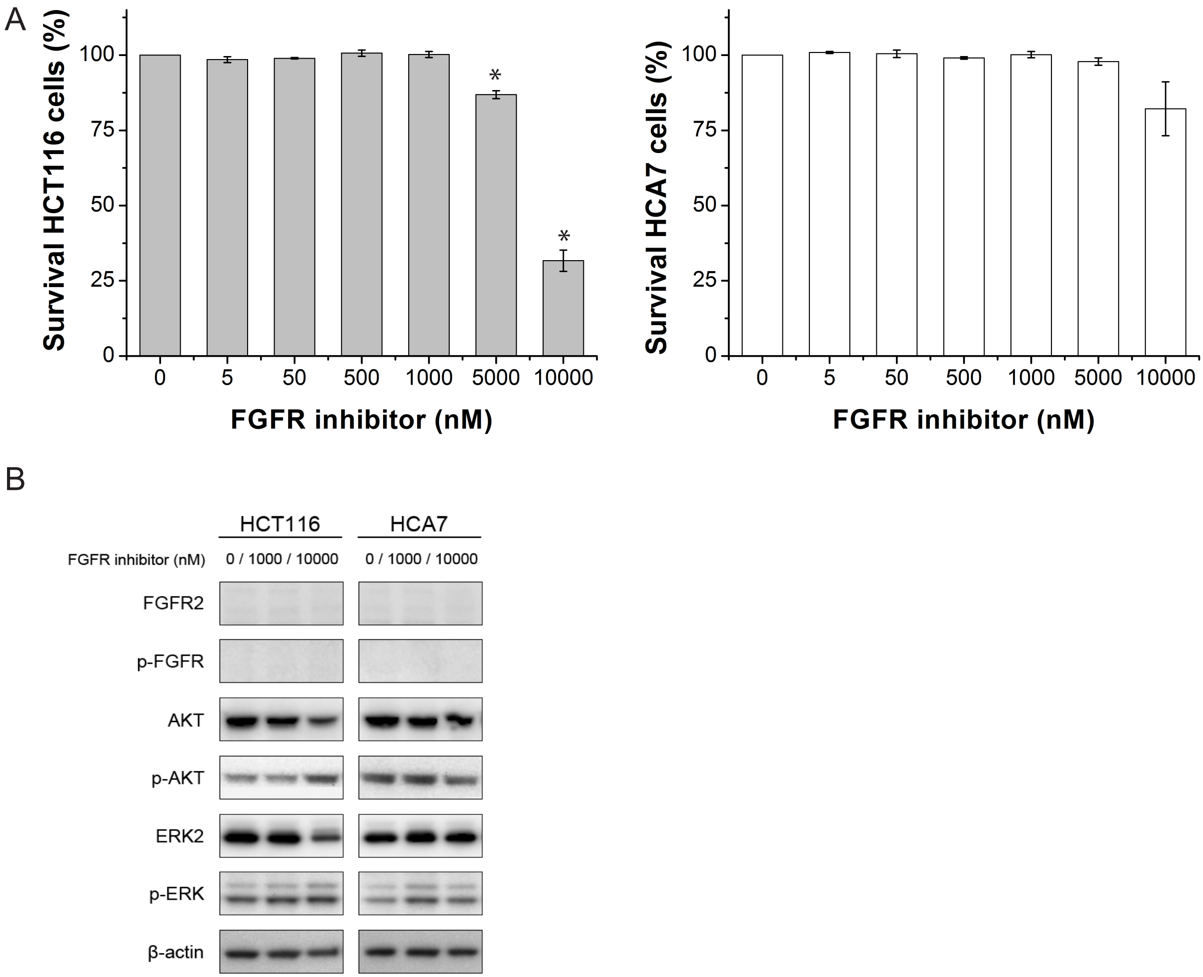

Figure e1. Effect on cell survival and protein levels. (A) Effect of different concentrations FGFR inhibitor for 72 hours incubation on cell survival determined by sulforhodamine B assay. Data = means ± SEM from three independent experiments performed in triplicate. *Significantly different from control conditions at the appropriate drug concentrations ($p < 0.05$; Tukey). (B) Immunoblot analysis of FGFR2 and downstream signaling molecules after 72 hours treatment. β-actin was used as a loading control. Blots shown are representative for two independent experiments.
