# Supplementary material for: In vitro and in vivo evaluation of the radiosensitizing effect of a selective FGFR inhibitor (JNJ-42756493) for rectal cancer
Source: BMC Cancer. 2015 Dec 16;15:946. doi: 10.1186/s12885-015-2000-8 (PMC4682227; doi:10.1186/s12885-015-2000-8)
Supplement: Additional file 2: Figure e2. — Effect on FGFR mRNA levels. (A) Quantification of FGFR mRNA expression in NCI-H716, Caco2, HCT116 and HCA7 cells after 72 h drug incubation. Data = means ± SEM of two independent experiments performed in duplicate. (B) Quantification of FGFR2 mRNA expression in NCI-H716 tumors isolated after drug treatment (TP1). Data = means ± SEM of three independent experiments. HPRT copy number was used to normalize the data. *Significantly different from control conditions at the appropriate drug concentrations (p < 0.05; Tukey). (PDF 4011 kb) [file 12885_2015_2000_MOESM2_ESM.pdf]

Figure e2

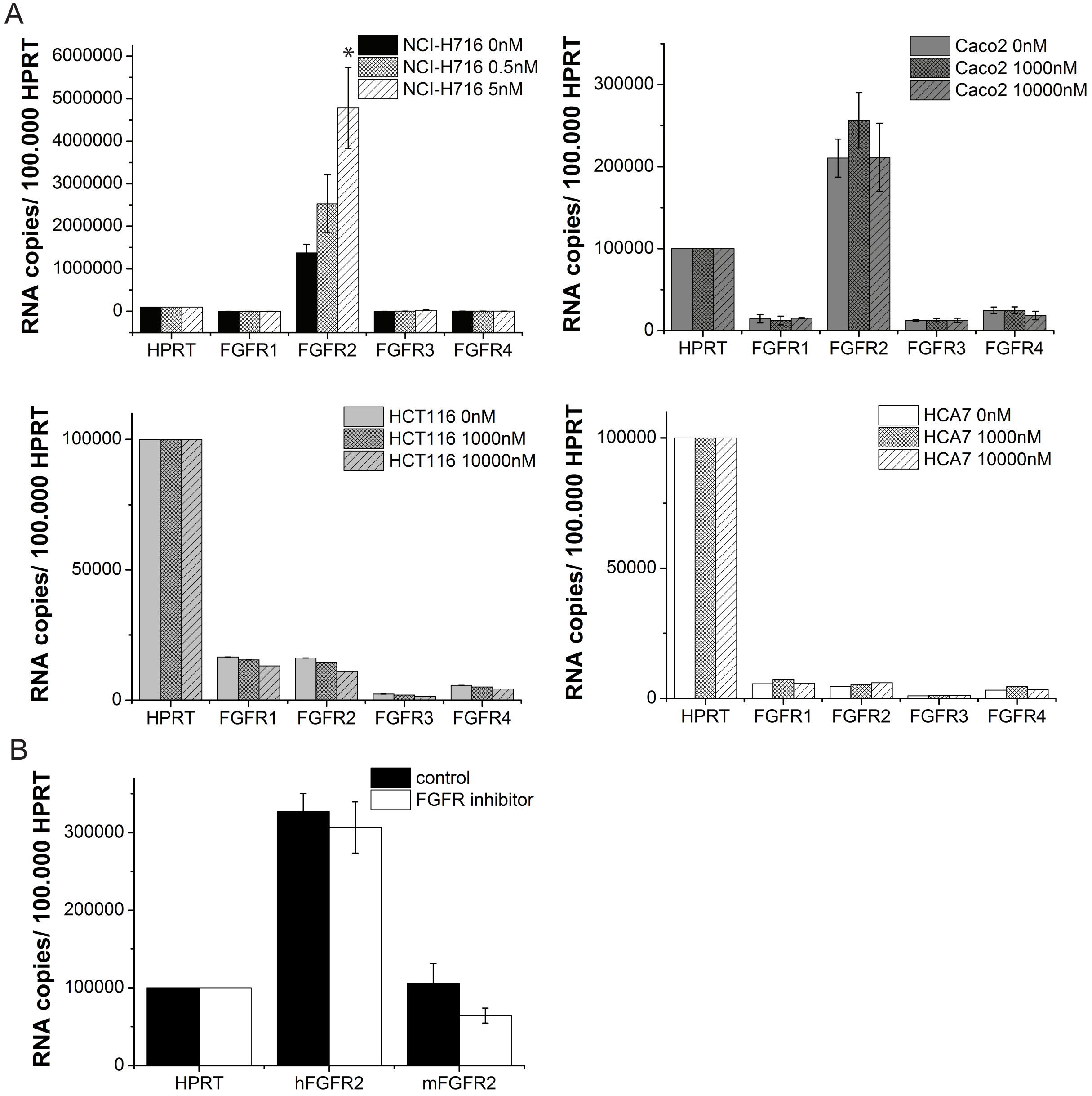

Figure e2. Effect on FGFR mRNA levels. (A) Quantification of FGFR mRNA expression in NCI-H716, Caco2, HCT116 and HCA7 cells after 72 hours drug incubation. Data = means ± SEM of two independent experiments performed in duplicate. (B) Quantification of FGFR2 mRNA expression in NCI-H716 tumors isolated after drug treatment (TP1). Data = means ± SEM of three independent experiments. HPRT copy number was used to normalize the data. *Significantly different from control conditions at the appropriate drug concentrations ($p < 0.05$; Tukey).
