# Supplementary material for: In vitro and in vivo evaluation of the radiosensitizing effect of a selective FGFR inhibitor (JNJ-42756493) for rectal cancer
Source: BMC Cancer. 2015 Dec 16;15:946. doi: 10.1186/s12885-015-2000-8 (PMC4682227; doi:10.1186/s12885-015-2000-8)
Supplement: Additional file 3: Table e1. — Primer sequences, primer probes and cycling conditions used for qPCR of different genes. (DOCX 15 kb) [file 12885_2015_2000_MOESM3_ESM.docx]

Table e1 Primer sequences, primer probes and cycling conditions used for qPCR of different genes

| ***In vitro*** | **Forward primer sequence** | **Reverse primer sequence** |
| --- | --- | --- |
| **FGFR1** | 5'-GACAGGTAACAGTGTCTGCTGA-3' | 5'-TGGAGGAGAGCCGTGAT-3' |
| **FGFR2** | 5'-TTCTTGGAGCCTGCACAC-3' | 5'-CGGGCTCGGAGGTATTC-3' |
| **FGFR3** | 5'-CCATCGGCATTGACAAGG-3' | 5'-GTCAGTGGCATCGTCTTTC-3' |
| **FGFR4** | 5'-TCTCCCGCTTCCCTCTG-3' | 5'-GAGACGCACGCCTCGTA-3' |
| **HPRT** | 5'-TGACACTGGCAAAACAATGCA-3' | 5'-GGTCCTTTTCACCAGCAAGCT-3' |
| ***In vivo*** | **Mouse probe** | **Human probe** |
| **FGFR2** | Mm01269930_m1 A&B | Hs01552918_m1 A&B |
| **FGF1** | Mm00438906_m1 A&B | Hs00265254_m1 A&B |
| **FGF2** | Mm00433287_m1 A&B | Hs00960934_m1 A&B |
| **VEGFA** | Mm00437306_m1 A&B | Hs00900055_m1 A&B |
| **PlGF** | Mm00435613_m1 A&B | Hs00182176_m1 A&B |
| **VEGFR1** | Mm01210866_m1 A&B | Hs.PT.45.1744053 NM_002019 IDT |
| **VEGFR2** | Mm01222419_m1 A&B | Hs00911700_m1 A&B |
| **HPRT** | Mm.PT.42.12662529 NM_013556 | Hs99999909_m1 A&B |
|  | **Cycling condition *in vitro*** | **Cycling condition *in vivo*** |
|  | 95°C 10:00 | 95°C 02:00 |
|  | 40 cycles 95°C 00:10, 60°C 00:30 | 40 cycles 95°C 00:03, 60°C 00:30 |
|  | 40°C 00:15 | 40°C 00:30 |
