# Supplementary material for: In vitro and in vivo evaluation of the radiosensitizing effect of a selective FGFR inhibitor (JNJ-42756493) for rectal cancer
Source: BMC Cancer. 2015 Dec 16;15:946. doi: 10.1186/s12885-015-2000-8 (PMC4682227; doi:10.1186/s12885-015-2000-8)
Supplement: Additional file 4: Table e2. — Specifications antigen retrieval, blocking step and antibodies used for immunohistochemical staining. (DOCX 15 kb) [file 12885_2015_2000_MOESM4_ESM.docx]

Table e2. Specifications antigen retrieval, blocking step and antibodies used for immunohistochemical staining

|  | **Ki67** | **Pimonidazole** | **Cleaved caspase-3** | **CD31** |
| --- | --- | --- | --- | --- |
| **Antigen Retrieval** | Tris EDTA, 20 min, microwave | 0.01M citrate buffer, 30 min, 95°C waterbath | Reveal buffer Decloaker (Biocare Medical, Concord, CA, USA) 30 min, 95°C waterbath | EDTA, 30 min, 95°C waterbath |
| **Blocking** | No | Protein block serum free, 30 min (DAKO, Glostrup, Denmark) | Protein block serum free, 10 min | 10% normal goat serum, 1% BSA, 0.3M glycine, 20 min + avidin-biotin (Vector Labs, Burlingame, CA, USA) |
| **Primary antibody** | 30 min, Thermo Scientific (Waltham, MA, USA) | 1/400, overnight 4°C, HP3-100kit rabbit antisera (Hypoxyprobe) | overnight 4°C, Biocare Medical | 1/100, overnight 4°C, Dianova (Hamburg, Germany) |
| **Secondary antibody** | 30 min, Envision secondary anti-rabbit labeled polymer/ horseradish peroxidase (HRP) conjugate (DAKO) | 30 min, Envision secondary anti-rabbit labeled HRP conjugate solution (DAKO) | 30 min, Envision secondary anti-rabbit labeled HRP conjugate solution (DAKO) | 1/100, 30 min, Goat anti-rat IgG (Vector Labs) + Vectastain ABC kit (Vector Labs) |
